# Case Study: Contribution of Extended Sequencing and Phylogeographic Analysis in the Investigation of Measles Outbreaks in Tunisia in 2019

**DOI:** 10.3390/vaccines12091085

**Published:** 2024-09-23

**Authors:** Anissa Chouikha, Marwa Arbi, Oussama Souiai, Henda Touzi, Zina Meddeb, Essia Ben Farhat, Mahrez Yahyaoui, Amel Ben Said, Chokri Hamouda, Henda Triki

**Affiliations:** 1 Laboratory of Clinical Virology, WHO Reference Laboratory for Poliomyelitis and Measles in the Eastern Mediterranean Region, Pasteur Institute of Tunis, University Tunis El Manar (UTM), Tunis 1002, Tunisia; touzihenda@yahoo.fr (H.T.); zinamedeb@gmail.com (Z.M.); henda.triki@pasteur.tn (H.T.); 2 Reasearch Laboratory “Virus, Vectors and Hosts: One Health Approach and Technological Innovation for a Better Health”, LR20IPT02, Pasteur Institute, University Tunis El-Manar (UTM), Tunis 1002, Tunisia; 3 Clinical Investigation Center (CIC), Institut Pasteur de Tunis, University Tunis El-Manar (UTM), Tunis 1002, Tunisia; 4 Laboratory of Bioinformatics, Biomathematics and Biostatistics, Institut Pasteur de Tunis, University of Tunis El-Manar (UTM), Tunis 1002, Tunisia; arbimarwa@gmail.com (M.A.); souiai@gmail.com (O.S.); 5 Higher Institute of Medical Technologies of Tunis, Tunis Al Manar University, Tunis 1068, Tunisia; 6 National Program of Immunization Basic Health Care Division, Ministry of Health Tunis, Tunis 1006, Tunisia; essia.hmida@gmail.com (E.B.F.); mahrezyahyaoui@gmail.com (M.Y.); amel3bensaid@gmail.com (A.B.S.); chokri.hamouda@fmt.utm.tn (C.H.)

**Keywords:** measles, N-450, MF-NCR, Tunisia, epidemic, genotyping, phylogeny, phylogeographic, variability

## Abstract

Despite the availability of an effective vaccine for several decades, the measles virus continues to spread worldwide. From 2018 to 2019, several countries experienced large measles outbreaks with genotype B3, including Tunisia. We analyzed 66 samples collected from serologically confirmed measles cases during this outbreak. Fifty-five percent were aged less than 12 months and had not received a measles vaccine. Phylogenetic analysis using the 450 nucleoprotein (N450) window revealed that all strains belonged to genotype B3, with five different variants identified. The N450 sequence of the predominant one, which circulated all through the epidemic period, was identical to the named strain MVs/Kabul.AFG/20.14/3. For better molecular discrimination, the amplification and sequencing of 1018 nucleotides in the non-coding region between the M and F genes (MF-NCRs) revealed higher variability with at least nine clusters. A phylogeographic study using Bayesian methods suggested the Governorate of Kasserine (on the borders of Algeria) as the introduction point with a TMRCA (Time to Most Recent Common Ancestor) for the 2019 sequences estimated around October 2018. These findings emphasize the crucial role of advanced molecular investigations in tracing measles transmission pathways which, together with good vaccine coverage, will help the final success of the global measles elimination program.

## 1. Introduction

Measles is a highly contagious, vaccine-preventable disease caused by the measles virus (MeV), a negative-sense RNA virus that belongs to the Morbillivirus genus within the *Paramyxoviridae* family [1]. The standard virus genome size is 15,894 nucleotides (nt) in length and contains six genes encoding different viral proteins: nucleoprotein (N), phosphoprotein (P), matrix protein (M), fusion protein (F), hemagglutinin (H), and polymerase (L). Each coding region is flanked by non-coding regions (NCRs) of variable length. The World Health Organization (WHO) recognizes 24 different MeV genotypes (A, B1-B3, C1-C2, D1-D11, E, F, G1-G3, H1, and H2) based on the nucleotide sequencing of the partial N and the H genes [2,3,4]. Since 1963, an effective and safe live-attenuated vaccine has been available and with efficacy against all circulating genotypes. However, measles remains a major cause of infant mortality in the developing world and continues to cause outbreaks in more developed countries [5,6]. In addition to existing gaps in immunization and surveillance programs, the COVID-19 pandemic caused service disruptions and the cancellation of planned supplementary immunization activities, further widening immunity gaps. In response, the WHO collaborated with various stakeholders in 2020 to establish the *Measles and Rubella Global Strategic Framework 2021–2030*, which aims to guide immunization stakeholders at the country, regional, and global levels in planning and implementing more effective measles and rubella elimination efforts, reinforcing the commitment to achieving a world free from these diseases [7].

As laboratory support is crucial for good surveillance, the WHO established the Global Measles and Rubella Laboratory Network (GMRLN) which consists of national, regional, and specialized laboratories that collaborate closely to achieve the global measles elimination goal. These laboratories follow standard procedures to confirm suspected measles cases, either serologically or by the molecular detection of the viral genome, and analyze the genetic characteristics of wild-type MeVs. The laboratories of the network convene annually in a global/regional meeting, are subject to accreditation, undergo yearly external quality control tests, and participate in frequent laboratory training sessions including the serologic and molecular confirmation of suspected cases as well as the genetic characterization of viruses from confirmed cases. MeV genotyping is performed by amplifying and sequencing a 450 nt fragment encoding the COOH terminus of the nucleoprotein according to the WHO manual [8,9,10,11]. In 2008, the WHO GMRLN established the Measles Nucleotide Surveillance (MeaNS) database to collect and archive measles virus sequences from all regions. Laboratories within the GMRLN use the MeaNS global database to submit their N450 nucleotide sequences and monitor the global circulation of measles genotypes [3]. Identical N450 sequences are represented by a Distinct Sequence Identification (DSId). Epidemiologically significant sequences that are circulating in several countries are defined as “Named strains” and are used to track transmission patterns for viruses within a single genotype. Circulating strains are linked to named strains which allows a finer mapping of measles transmission than the use of genotyping alone [3].

In recent years, the only reported MeV genotypes are B3 and D8, while the other genotypes have not been detected. This is due to the significant increase in vaccination coverage, which has greatly reduced the diversity of circulating measles viruses [7]. As genetic diversity decreases, the standard N450 sequences may show very low diversity during large outbreaks involving one or multiple countries, with identical sequences. Furthermore, when monitoring measles transmission within a country or region over time, N450 sequences generally do not enable differentiation between ongoing endemic circulation of a MeV variant and multiple new introductions [4,12,13]. To enhance the resolution of molecular epidemiology analysis, especially in this final phase of elimination, sequencing a larger fragment in other hypervariable genomic regions has been proposed. The MF-NCR, located between the fusion and matrix genes, is one of the most variable regions of the measles virus genome and has shown the better resolution of the virus circulation patterns. It is now proposed as a new target for MeV molecular characterization [13,14,15,16,17].

In Tunisia, the measles vaccine was introduced into the national program of immunization in 1983 with two doses of the vaccine given at varying ages: 9 months and 15 months from 1983 to 1998; 15 months and 6 years from 1999 to 2012; and 12 months and 18 months starting from 2013. The vaccination coverage has achieved levels over 90% nationwide over the last decade and, as a results, measles incidence has considerably decreased. The last large outbreak of measles occurred in 1992 with more than 11,000 cases [18]. A measles/rubella national elimination goal was established in 1998 [17]. Since then, few serologically confirmed measles cases have been reported, and are generally less than 20 cases per year except in 2002 (72 confirmed cases) and in 2012 (48 confirmed cases) [18,19].

In 2019, together with many other countries in the world, Tunisia experienced a nationwide measles outbreak [19,20] with a total of 4698 measles cases, among which 1208 were serologically confirmed, 1174 were epi-linked, and 2316 were clinically compatible. The outbreak also resulted in 39 deaths, most of them occurring in infants of less than one year old or in adults suffering from immunodeficiency or other debilitating illnesses [21].

Concurrently, outbreaks occurred in nearby countries, notably Libya and Algeria, as well as in France and Italy, which have substantial population movements with Tunisia. This raised the possibility of virus importation at the origin of the outbreak. The initial cases were detected in two governorates: Kasserine, located in the west near the Algerian border, and Sfax, on the east coast, which frequently receives people from Libya and Algeria for healthcare services. During the 2019 outbreak, Sfax and Kasserine were the most affected areas of the reported cases. However, no epidemiological evidence of importation from Libya to Sfax or from Algeria to Kasserine was found and no clear epidemiological link could be established between the cases in the two Tunisian governorates.

The aim of this study is to assess the genetic variability of MeVs strains from patients infected during the 2019 outbreak in Tunisia and to study the dynamics of virus spread during the outbreak by analyzing the N450 and MF-NCR sequences.

## 2. Materials and Methods

### 2.1. Clinical Specimens

This study was conducted on 88 samples collected from 88 positive cases of serologically confirmed measles (n = 48, throat swab; n = 39, oral fluid; and n = 1, urine). The samples were collected from suspected measles cases in Tunisia between January and December 2019 and were stored at the Laboratory of Clinical Virology in Pasteur Institute of Tunis, which serves as the national laboratory for MeVs detection and genotyping and as a WHO regional reference laboratory in the eastern mediterranean region (EMR).

### 2.2. PCR Amplification and Sequencing

For PCR amplification and sequencing, we used US-CDC RT-PCR methods, as outlined in the WHO lab manual [8]. Viral RNA was extracted using QIAamp viral RNA mini Kit (Qiagen, Hilden, Germany) from 140 µL of clinical specimens, according to the manufacturer’s instructions. The extracted RNAs were assessed for virus detection by real time RT-PCR [8] and for genome amplification were reverse transcribed and then amplified by PCR in the nucleoprotein and the MF-NCRs of the genome. In the nucleoprotein region, a 634 nucleotide-long (nt) fragment in the 5′ terminus of the nucleocapsid protein gene was amplified using WHO-recommended primers (MeV216-Forward: 5′-TGG AGC TAT GCC ATG GGA GT-3′; MeV214-Reverse: 5′-TAA CAA TGA TGG AGG GTA GG-3′) provided by CDC-USA. In the MF-NCR, a fragment of 1018 nt was deduced for each samples from two overlapping fragments amplified using the following primers, also provided by US-CDC: fragment 1 (MeV4331-Forward: 5′-CAG ATG CAA GAT AGT AAG AAT CCA G-3′; V4869-Reverse: 5′-CCT GGC CCT CAG TTT TGT TTA G-3′) and fragment 2 (MeVg22-Forward: 5′-CAC AAG CGA CCG AGG TGA C-3′; MeV5145R-Reverse: 5′-GGT TGC CGT GGT CGT GTG TG-3′).

RT-PCR was performed using the One-Step RT-PCR kit (Invitrogen Superscript III One-step RT-PCR with Platinum, Canada) using the CDC-USA standard protocol. In a total volume of 50 µL containing 5 µL extracted RNA, 0.6 μM of each primer, and 0.4 μM of dNTPs were used. The amplification conditions included an initial reverse transcription step of 30 min at 50 °C, followed by a denaturation step of 15 min at 95 °C then by 40 cycles of 95 °C for 30 s, 55 °C (N-gene)/56 °C (MF-NCR) for 30 s, and 72 °C for 30 s, and a final extension step was performed at 72 °C for 10 min.

Amplicons were then purified and sequenced, in both directions, with the ABI Big Dye Terminator Cycle Sequencing Kit (Applied Biosystems, Branchburg, NJ, USA) using the same primers described above and used for PCR amplification.

### 2.3. Sequence Analyses and Phylogeny

The N-450 and MF-NCR sequences of each virus strain were obtained by aligning the forward and reverse sequences using Sequencher^®^ 5.1.4 software. The N450 sequences were submitted to the Measles Nucleotide Sequencing database (MeaNS) and the MF-NCR sequences were submitted to GenBank under accession numbers MT211788-MT211848.

Sequence alignment was performed using MEGA software version 7 and default parameters. The resulting alignment was used to build a maximum likelihood phylogenetic tree using the IQ-TREE web server, supported by 1000 bootstrap replicates (http://iqtree.cibiv.univie.ac.at/) (accessed on 18 May 2024). Midpoint rooting was used for visualization using Figtree software (http://tree.bio.ed.ac.uk/software/figtree/) (accessed on 18 May 2024). The tree was rooted using the midpoint rooting method.

### 2.4. Phylogeography Analysis

Bayesian phylogeography was performed by generating a time-scaled Maximum Clade Credibility (MCC) phylogenetic tree using BEAST v.1.8.4 package. The Bayesian Markov Monte Carlo chain was run for 300 million iterations (sampling every 10,000 states) using the uncorrelated relaxed clock and the skyline tree models. A Bayesian stochastic search variable selection (BSSVS) with a symmetrical discrete trait substitution model and a strict clock assumption were implemented to construct the Bayes factor test and were used to identify the transmission routes between Tunisian regions [22].

The most important parameter estimates (Posterior, Prior, Kappa, clock rate, TMRCA, TreeModel.rootHeight, treeLength, treeLikelihood, and Gamma shape) exhibited a valid effective sampling size (ESS > 200). The MCC tree was generated by Tree Annotator v1.8.4 after removing 10% burn-in and was then visualized in FigTree v.1.4.3 (http://tree.bio.ed.ac.uk/software/figtree/) (accessed on 18 May 2024). The Time To the Most Recent Common Ancestor (TMRCA) and its 95% highest posterior density (95% HPD) was estimated using FigTree v.1.4.3. A posterior probability (pp) of >0.85 means that the MCC tree node has good statistical support [23]. Bayes factors for discrete state transitions were calculated with SpreadD3map of Google Earth Pro (https://www.google.com/earth/versions/) (accessed on 18 May 2024). The Bayes Factor was also calculated with SpreadD3. BF > 3 indicates statistical support for the transitions.

## 3. Results

### 3.1. Epidemiological Data

Of the 88 samples from serologically confirmed cases included in the study, 66 were amplified in the N450 region, among which 61 were also amplified in the MF-NCR. The remaining 22 samples were not amplified in both genomic regions. Their Ct values obtained from the real-time PCR tests were over 34, which indicates a low viral load. The 66 cases comprised 35 females and 31 male, aged 1 month to 62 years. Thirty-six patients were under one year old and were thus unvaccinated, ten were infants aged between 1 and 13 years, and twenty were adults aged between 24 and 62 years.

**Figure 1:**
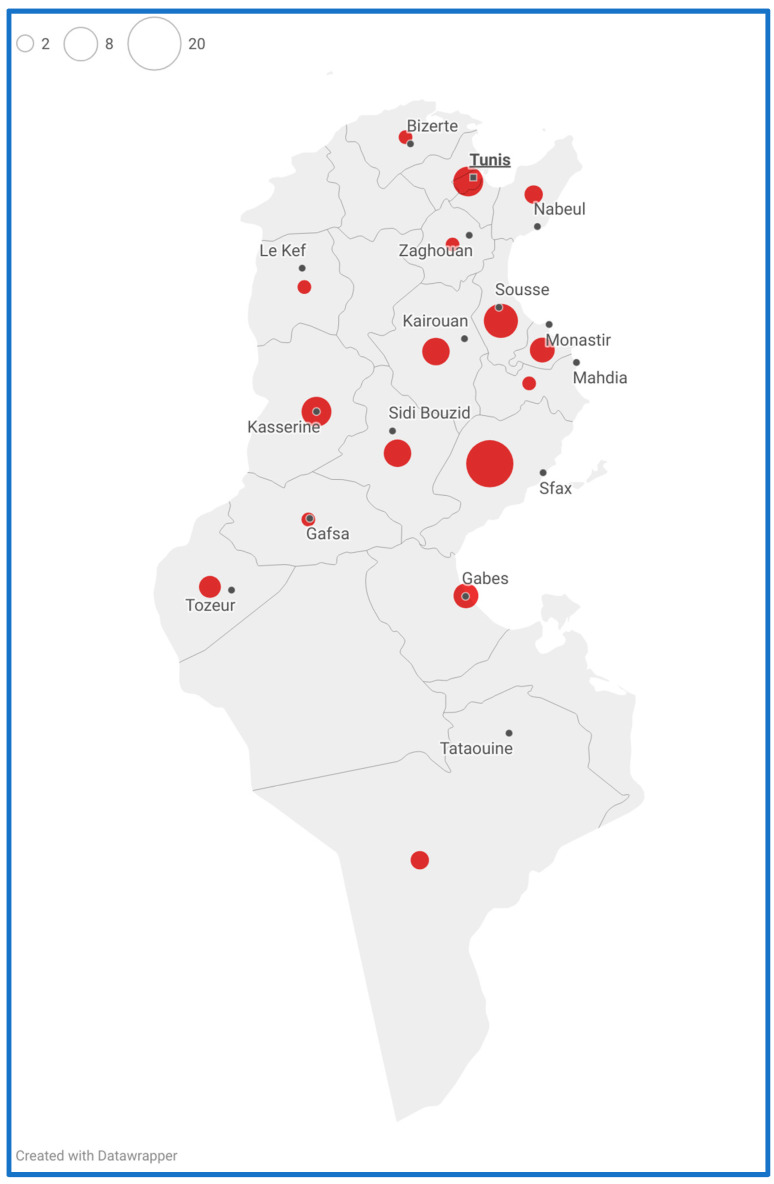
Distribution of the 66 genotyped measles cases during the 2019 outbreak in Tunisia by governorate. The map was created with Datawrapper (https://www.datawrapper.de/) (accessed on 18 May 2024). The size of the circles on the map correspond to the number of cases; larger circles indicate a higher number of cases.

The studied samples originated from almost all the governorates of Tunisia; only four governorates (Béjà, Jendouba, Medenine, and Siliana) were not represented. These governorates were the less affected by the outbreak, accounting, together, for almost 1% of the nationally recorded measles cases. Figure 1 shows their distribution by date of sample collection together with the total number of suspected cases recorded by the Ministry of Health. The 66 cases were spread across almost the entire outbreak period. Figure S1 shows their distribution by date of sample collection during the outbreak period.

### 3.2. Measles Virus Genotyping

A measles virus genotype was successfully determined by the sequencing of the N450 region of the 66 studied samples obtained from measles-positive cases.

**Figure 2:**
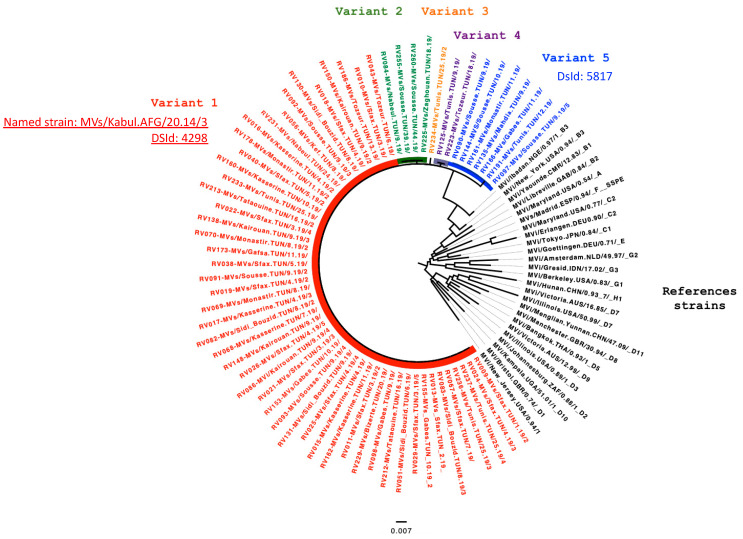
Phylogenetic tree of 66 measles virus N450 sequences from Tunisia (2019) compared with the 28 reference sequences. This tree compares 66 N450 sequences of measles viruses collected in Tunisia from week 1 to week 44 in 2019 with 28 reference sequences representing the 24 genotypes (each of the genotypes B3, C2, D5, and D7 are represented by 2 reference sequences). The tree highlights five different variants: Variant 1 (DSId: 4298) (in red) comprises fifty sequences; Variant 2 (DSId: 5847) (in green) comprises four sequences; Variant 3 (DSId: 6067) (in orange) comprises one sequence; Variant 4 (DSId: 5846) (in purple) comprises two sequences; and Variant 5 (DsId: 5817) (in blue) comprises seven sequences. The sequence names are formatted as Lab code–WHO name. The tree was constructed using the maximum likelihood method via the IQ Tree web server and was visualized using FigTree. The topology is supported by 1000 bootstrap replicates.

Figure 2 compares the Tunisian sequences with the WHO reference sequences representative of all measles genotypes. The phylogenetic analysis revealed that all Tunisian sequences belonged to genotype B3, according to the topology of the phylogenetic tree shown in Figure 2.

**Figure 3:**
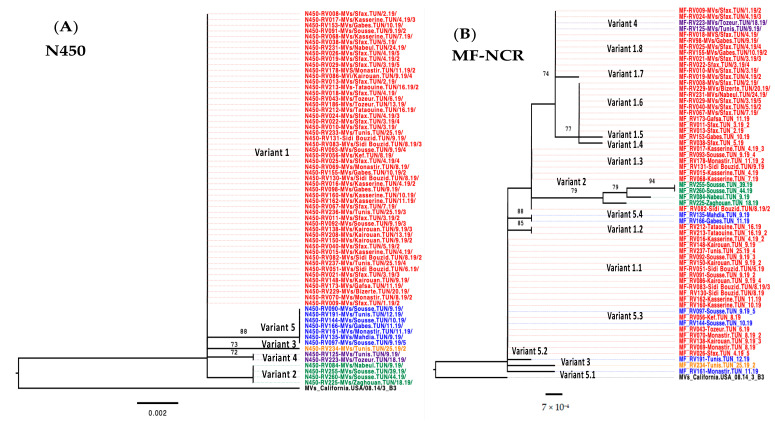
Phylogenetic trees comparing the N450 and the MF-NCR sequences of 66 vs. 61 Tunisian sequences, respectively. (**A**) Sequences representing the 450 nt in the C-terminal nucleoprotein gene of the measles genome. (**B**) Sequences representing 1018 nt in the non-coding MF-NCR of the measles genome. The trees were constructed using the maximum likelihood method via the IQ Tree web server and were visualized using FigTree. The topology was supported by 1000 bootstrap replicates.

The most frequently detected variant (Variant 1) represented in red in Figure 3 comprises 50 sequences identical to the named strain MVs/Kabul.AFG/20.14/3 with the Distinct Sequence ID (DSId: 4298). As of 20 May 2024, this strain is widely distributed across 27 countries on 5 continents. The sequences for this variant were obtained from patients from 14 different governorates in Tunisia.

According to the MeaNS database exact match analysis, no named strain was associated with the sequences of the other four variants. The sequences of Variant 5 (DsId: 5817), shown in blue, originated from four governorates on the eastern coast of Tunisia and were identical to the measles sequences detected in Valence, France, in early 2019.

The sequences from the remaining three Variants 2, 3, and 4 were new and had no identical matches in the MeaNS database with DSIds: 5847, 6067, and 5846, respectively.

Low variability was observed between the different variants in the N450 region as follows: Variants 2 and 4 differed from Variant 1 by one mutation in nucleotides 285 and 312, respectively; Variants 3 and 5 differed from Variant 1 by two mutations in nucleotides 57 and 176 and 13 and 87, respectively.

### 3.3. Phylogenetic Analyses in the MF-NCR

The MF-NCRs of the 61 Tunisian samples were successfully amplified and sequenced. All sequences were 1018 nt in length with no insertions or deletions detected.

The phylogenetic analysis showed a higher genetic variability as compared to the N-450 region, with at least 13 different clusters (Figure 3). The sequences from the major N450 Variant 1 were divided into eight different clusters in the MF-NCR. The sequences obtained from N450 Variant 5 (in blue) were divided into four different clusters in the MF-NCR. The sequences obtained from the N450 Variants 2, 3, and 4 remained together in the MF-NCR (Figure 3).

**Figure 4:**
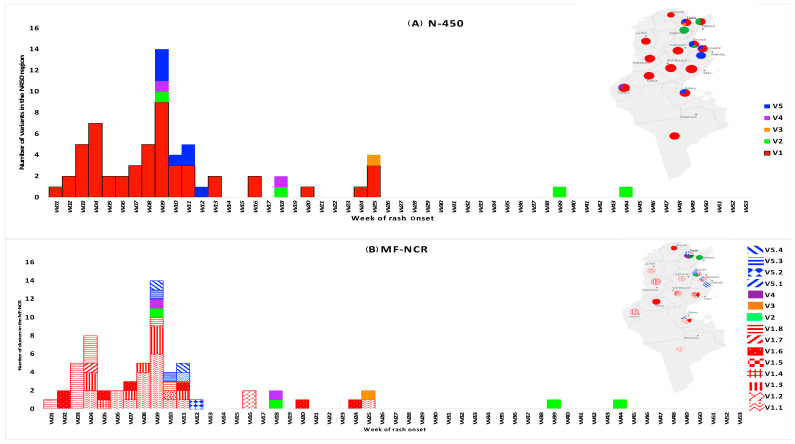
Timeline and origin of detection of the different measles virus variants identified in the N450 region (**A**) and in the MF-NCR (**B**), Tunisia 2019.

Figure 4 shows the circulation of the different variants in both N450 (Figure 4A) and MF-NCR (Figure 4B) during the outbreak period. The N450 Variant 1 (red) circulated from week 1 to week 25, suggesting that the same strain would have circulated throughout the outbreak. Concurrently, analysis of the MF-NCR revealed that this variant actually consisted of eight different variants during the same period, all sharing the exact same N450 sequence. For example, Subvariant 1.8 (Figure 4B) circulated from week 1 to week 10, Subvariant 1.6 circulated from week 2 to week 24, and Subvariant 1.1 circulated from week 4 to week 25.

A similar pattern was observed with the N450 Variant 5 (blue), which divided into four distinct variants in the MF-NCR (Figure 3 and Figure 4). This variant was detected from week 9 to week 12. For Variant 3, represented in orange, only one sequence was detected in week 25, while two sequences were identified for Variant 4, represented in purple, detected sporadically in weeks 9 and 18; these sequences clustered alone in both the N450 and the MF-NCR. The N450 Variant 2 (green) included four identical N450 sequences that clustered together in the MF-NCR, despite the few mutations among them. This latter variant would have been circulating at least from week 9 to week 44.

### 3.4. Spatio-Temporal Dynamics of Measles Virus Circulating in Tunisia in 2019

**Figure 5:**
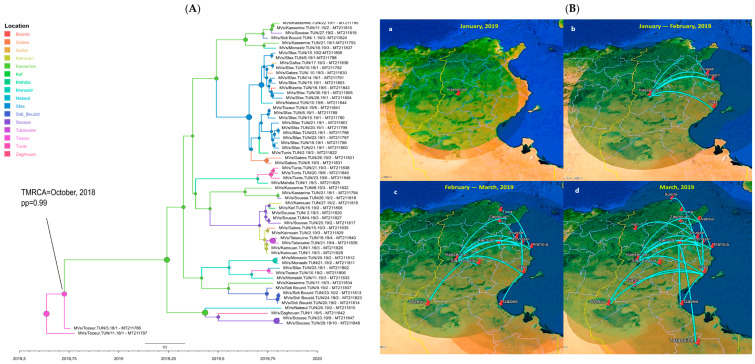
The phylodynamics of the measles virus detected in Tunisia in 2019 based on the MF-NCR. (**A**) Bayesian time-scaled MCC tree of the measles virus isolated from Tunisian governorates. The nodes and branches of the MCC tree are colored to illustrate the relevant Tunisian governorates. Node diameters are sized according to posterior probabilities. (**B**) Spatio-temporal dynamics of the B3 measles virus that circulated in Tunisia in 2019 among different Tunisian governorates. The maps (**a**–**d**) illustrate the various stages of virus spread across the governorates. Transition lines connecting different locations represent the branches in the MCC tree. The diameters of the circles are proportional to the square root of the number of MCC branches that maintain a particular location state at each time point.

From the dataset composed of the 61 measles MF-NCR sequences (1018 nt), the MCC (Maximum Clade Credibility) tree was built and used to explore the temporal origin of the selected dataset by determining the TMRCA (Time to Most Recent Common Ancestor) (Figure S1). According to the molecular clock analysis, the TMRCA for the measles virus that circulated in Tunisia in 2019 was estimated to be around October 2018 (pp = 0.99) (Figure 5A). For N450, the TMRCA estimation showed that the first introduction of measles virus in Tunisia was in April 2019 (pp = 0.95) (Figure S2.1).

To investigate the spatio-temporal dynamics of the B3 measles spread in Tunisia in 2019, a Bayesian phylogeographic framework based on MF-NCR sequences was constructed (Figure 5B). Figure 5(Bb) illustrates the routes of transmission of the epidemic measles viruses from where the outbreak started and how it spread across the country. The generated spatial dynamics confirmed that the initial point of propagation was the governorate of Kasserine (center west of the country), located on the border with Algeria. From Kasserine, the virus spread to other Tunisian governorates (Figure 5(Ba)).

During the January–February period 2019, the virus emerged in the coastal governorates, notably Sousse (BF = 15.59), Monastir (BF = 53), and Sfax (BF = 3.46) (Figure 5(Bb)). By February–March 2019, the epidemic extended further, reaching all governorates, namely in the northern (Zaghouan, Nabeul, Tunis, Kef), in the central (Sidi Bouzid, Kairouan), and in the southern regions (Gafsa, Gabes, Tozeur, Tataouine) of Tunisia (Figure 5(Bc,Bd)). Most of these transitions were statistically supported with BF > 3 (Sfax–Nabeul: BF = 18.05; Kasserine–Sidi Bouzid: BF = 16.07; Sfax–Gafsa: BF = 10.76; Sfax–Gabes: BF = 230.21; Sfax–Tozeur: BF = 12.54).

Based on the N450 analysis, spatio-temporal dynamics revealed that the epidemic of measles virus in Tunisia started from Tunis and spread to Sousse (BF = 5.86) in May 2019 (Figure S2.2A). In July 2019, the virus emerged in the coastal regions (Monastir and Gabes). All these transitions had statistical support (Sousse–Monastir: BF = 4.97; Sousse–Gabes: BF = 7.27) (Figure S2.2B). Then, the transmission zone extended to reach other coastal regions (Mahdia and Sfax) as well as the center of Tunisia (Sidi Bouzid, Kairouan and Kasserine) (Figure S2.2C). Only the transition linking Sousse to Mahdia was supported by a BF equal to 7.94. In October, a total emergence of the virus was observed in many regions in the north (Zaghouan, Nabeul, Bizerte and Kef) and south of Tunisia (Tataouine, Tozeur) (Figure S2.2D). Here, only the Sousse–Zaghouane (BF = 7.45) and Tunis–Tozeur (BF = 30.76) transitions were statistically supported.

## 4. Discussion

In the present work, we found that all the epidemic sequences from Tunisia belong to the B3 genotype. According to the N450 genomic region, most of the sequences (76%, 50 out of 66) belonged to one major variant related to the MVs/Kabul.AFG/20.14/3 named strain (DsId: 4298), which circulated in 17 out of 24 affected governorates of the country and during almost the whole epidemic period, lasting from week 1 to week 25. The N450 phylogenetic analyses identified four other minor variants; one of them was detected in Valence, France, in 2019 and the three others are new and are not yet reported in the MeaNS database. Thus, according to the N-450 genomic region, these minor variants may represent different individual importations while the major variant would be the main virus strain responsible for the outbreak.

We undertook heavy sampling in the early outbreak period and less sampling in the later stages, which may constitute a potential limitation to the accuracy of our findings.

When using the MF-NCR, the findings were clearly different, since more sets of identical sequences were identified. The sequences from the N450 major cluster split into eight subvariants each, with one detected during a shorter period of time.

The use of new sequencing windows, including the MF-NCR as well as whole genome sequencing, has been recommended for improving measles surveillance [15]. A scan of sequence variability across the full MeV genome identified the MF-NCR as the most variable part of the MeV genome [14,15,16]. The MF-NCR comprises 426 nucleotides of the matrix (M) protein gene its 3′ end, followed by an intergenic region of 3 nucleotides and then 583 nucleotides of the fusion (F) protein gene in its 5′ end. It is the longest non-coding region of the measles virus genome; it is rich in G-C and its functionality is not well understood [17,24]. Previous studies used this genomic region and a found better resolution between measles strains that circulated in concomitant outbreaks or over several years in one country, consistent with epidemiological data [14,15]. However, additional studies in other endemic or epidemic contexts, such as the present work, are needed to better demonstrate the added value of MF-NCR in the molecular investigation of measles transmission. Also, while a large number of sequences are available for the N and the H genes for all genotypes of the measles virus, there are still far fewer sequence data for the MF-NCR. The further identification of MF-NCR sequences from other regions of the world is essential to broaden the number of sequences used in phylogenetic analyses and study transmission patterns at national and international contexts, and our work adds new sequence data from the EMR and north African region.

From the technical side and in the present work, we were able to amplify the MF-NCR of 61 out of 66 samples that showed positive amplification in the N-450 region. Previous authors were also able to obtain MF-NCR sequences from a high proportion of samples that were positive in the N gene region despite the high genetic variability in this region [14]. We did not find deletions or insertions in our B3 MF-NCR sequences, as found in the MF-NCRs of other genotypes [2,11,25,26]. Our results add arguments on the potential easy use of this region as a complementary tool for epidemiological investigation.

The first case of the outbreak was from Kasserine; it was serologically confirmed but no samples for virus detection were collected. Subsequently, several cases were identified in the same governorate and all of these were either serologically confirmed, epi-linked, or clinically confirmed, but no samples for virus detection were collected. The first cases in Kasserine where oral fluid samples were collected had a rash onset on 21st January 2019 and after. On the other hand, the cases identified in Sfax in the same period and starting at the beginning of the outbreak had oral fluid or throat swabs samples collected immediately. The possibility of two routes of transmission was, in fact, raised, but our phylogeographic analyses suggested only one source of transmission, starting from Kasserine. This underscores the value of phylogeographic analyses based on genomic sequences, as the analysis was able to trace the origin of the infection despite the relative delay in obtaining samples from certain regions and the lack of clear epidemiological links between cases as generally provided by the classical epidemiological investigations. The GMRLN does not currently mandate the sequencing of this genomic region; however, many laboratories within the network with sequencing capacities are tracking MeV transmission pathways using MF-NCR sequences. In the present study, we investigated the phylogenetic and phylodynamic analysis of the MF-NCR of the MeV detected in Tunisia during the outbreak of 2019. The phylodynamic study based on the MF-NCR suggests the virus’ actual pathways during the outbreak, including the initial point of spread and its subsequent dissemination in the country. Indeed, based on the dates of case notifications to the Ministry of Health, the first cases were from Kasserine with concomitant cases in Sfax starting in week 1, 2019. The notifications started to come from other governorates, and by week 13, the whole country was affected. Our findings align with these epidemiological data as, based on the sequence analysis, the spread of the disease reached all the governorates from which we obtained virus sequences in March. In addition, given the lack of epidemiological link between the cases from different locations, our analyses provide information on the possible routes of transmission from one governorate to another. This outcome aligns with epidemiological information.

Previous phylogenetic and phylodynamic analyses conducted by Jacqueline et al. (2023) and Kim et al. (2021) using MF-NCR sequencing showed that it provided enhanced resolution while tracing the epidemiological routes of measles outbreaks in Spain and South Korea, respectively [27,28]. Our MF-NCR analyses align with epidemiological information based on case notification dates, dates of rash onset, and geographical origin for the measles virus spread. However, we could not perform the analyses for each variant separately as higher numbers of sequences from each variant were needed, and this is one of the limitations of this study. Another limitation is the frequency of sample collection during the outbreak with a lower number of samples collected starting from April and this represents a potential limitation to the accuracy of our phylogenetic analyses.

Our study relies on self-reported data, which may be subject to sampling bias. This could limit the accuracy of the phylogenetic methods, which is due particularly to the unequal distribution of sample sizes over various times and locations.

## 5. Conclusions

In conclusion, this study provides critical baseline data on the wild-type measles viruses that caused the Tunisia outbreak in 2019, which will be very useful for supporting measles elimination in the future. Our study adds evidence that, in addition to sequencing in the N gene, sequencing in the MF-NCR enhances the differentiation of epidemic strains and adds some value towards recommending this supplemental sequencing.

## Data Availability

The original contributions presented in the study are included in the article/Supplementary Materials, further inquiries can be directed to the corresponding author chouikhaanissa@gmail.com.

## Supplementary Materials

The following supporting information can be downloaded at: https://www.mdpi.com/article/10.3390/vaccines12091085/s1, Figure S1: Distribution by week of rash onset of the number of the 66 genotyped measles cases (Histogram in red) and of the number of Measles cases notified to the ministry of health (blue curve) during the MeV outbreak in Tunisia 2019; Figure S2: Phylodynamics of Measles virus detected in Tunisia in 2019 based on N-450 genomic region. 1. Bayesian Time scaled MCC tree of Measles virus isolated from Tunisian governorates. The nodes and branches of MCC tree are colored to express the inferred Tunisian governorates. Node diameters are sized according to posterior prbabilities. 2. Spatio-temporal dynamics of the B3 measles virus that circulated in Tunisia in 2019 among different Tunisian governorates. The snapshots (A–D) illustrate the various stages of virus spread across the governorates. Transition lines connecting different locations represent the branches in the MCC tree. The diameters of the circles are proportional to the square root of the number of MCC branches maintaining a particular location state at each time point.
