# Supplementary material for: Case Study: Contribution of Extended Sequencing and Phylogeographic Analysis in the Investigation of Measles Outbreaks in Tunisia in 2019
Source: Vaccines (Basel). 2024 Sep 23;12(9):1085. doi: 10.3390/vaccines12091085 (PMC11435752; doi:10.3390/vaccines12091085)
Supplement: Supplementary file 1 [file vaccines-12-01085-s001.zip › Figure S1.pdf]

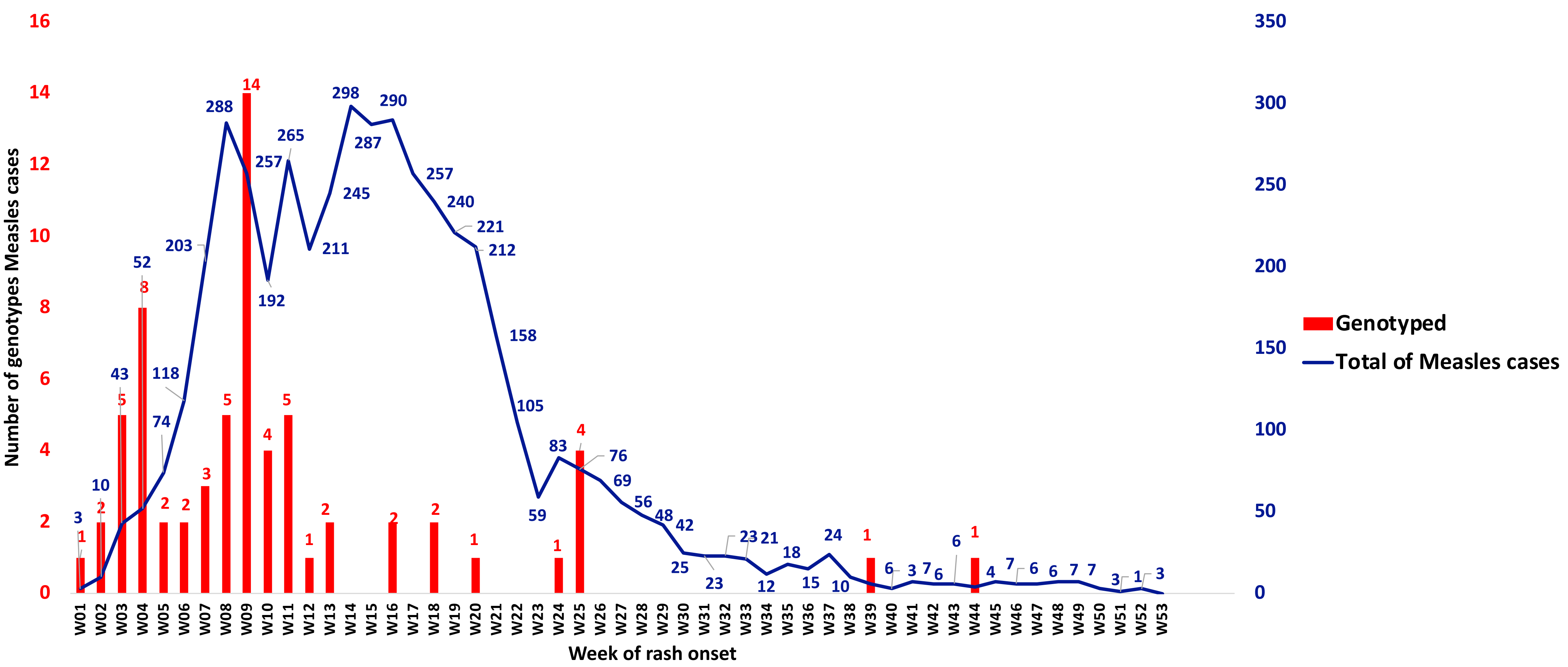

Figure S1. Distribution by week of rash onset of the number of the 66 genotyped measles cases (Histogram in red) and of the number of Measles cases notified to the ministry of health (blue curve) during the MeV outbreak in Tunisia 2019.
