# Supplementary material for: Case Study: Contribution of Extended Sequencing and Phylogeographic Analysis in the Investigation of Measles Outbreaks in Tunisia in 2019
Source: Vaccines (Basel). 2024 Sep 23;12(9):1085. doi: 10.3390/vaccines12091085 (PMC11435752; doi:10.3390/vaccines12091085)
Supplement: Supplementary file 1 [file vaccines-12-01085-s001.zip › Figure S2.pdf]

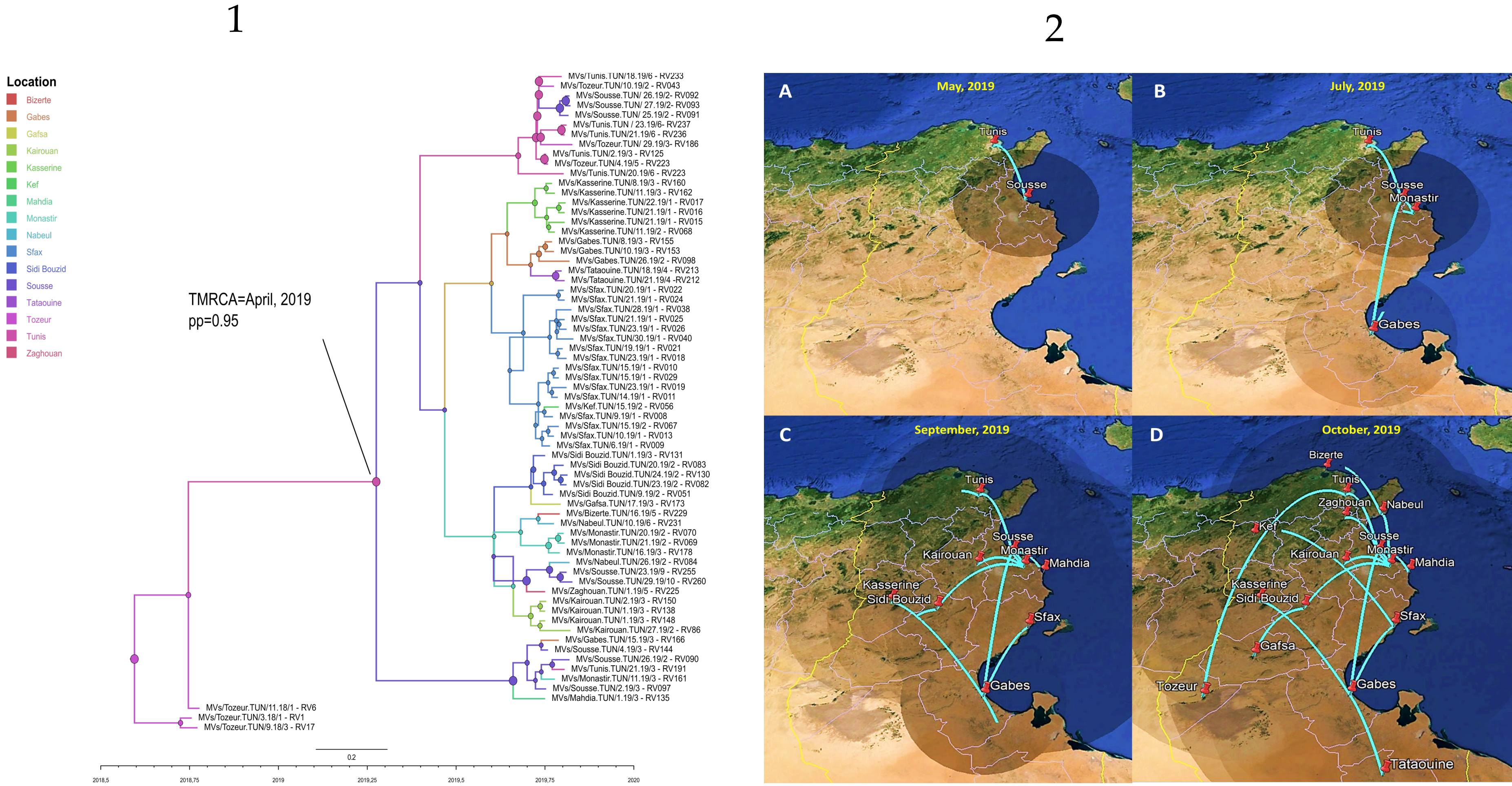

Figure S2: Phylodynamics of Measles virus detected in Tunisia in 2019 based on N-450 genomic region. 1. Bayesian Time scaled MCC tree of Measles virus isolated from Tunisian governorates. The nodes and branches of MCC tree are colored to express the inferred Tunisian governorates. Node diameters are sized according to posterior prbabilities. 2. Spatio-temporal dynamics of the B3 measles virus that circulated in Tunisia in 2019 among different Tunisian governorates. The snapshots (A–D) illustrate the various stages of virus spread across the governorates. Transition lines connecting different locations represent the branches in the MCC tree. The diameters of the circles are proportional to the square root of the number of MCC branches maintaining a particular location state at each time point.
